# An agent-based modeling framework for the design of a dynamic closed-loop supply chain network

**DOI:** 10.1007/s40747-022-00780-z

**Published:** 2022-06-28

**Authors:** Ayşegül Bozdoğan, Latife Görkemli Aykut, Neslihan Demirel

**Affiliations:** 1 Department of International Trade and Logistics, Faculty of Applied Sciences, Kayseri University, 38280 Kayseri, Turkey; 2 Department of Industrial Engineering, Faculty of Engineering, Erciyes University, 38039 Kayseri, Turkey

**Keywords:** Closed-loop supply chain (CLSC), Network design, Agent-based modeling (ABM), Customer behavior, AnyLogic

## Abstract

The supply chain is a dynamic and uncertain system consisting of material, information, and fund flows between different organizations, from the acquisition of the raw materials to the delivery of the finished products to the end customers. Closed-loop supply chains do not end with the delivery of the finished products to the end customers, the process continues until economic value is obtained from the returned products or they are disposed properly in landfills. Incorporating reverse flows in supply chains increases the uncertainty and complexity, as well as complicating the management of supply chains that are already composed of different actors and have a dynamic structure. Since agent-based modeling and simulation is a more efficient method of handling the dynamic and complex nature of supply chains than the traditional analytical methods, in this study agent-based modeling methodology has been used to model a generic closed-loop supply chain network design problem with the aims of integrating customer behavior into the network, coping with the dynamism, and obtaining a more realistic structure by eliminating the required assumptions for solving the model with analytical methods. The actors in the CLSC network have been defined as agents with goals, properties and behaviors. In the proposed model dynamic customer arrivals, the changing aspects of customers' purchasing preferences for new and refurbished products and the time, quantity and quality uncertainties of returns have been handled via the proposed agent-based architecture. To observe the behavior of the supply chain in several conditions various scenarios have been developed according to different parameter settings for the supplier capacities, the rate of customers being affected by advertising, the market incentive threshold values, and the environmental awareness of customers. From the scenarios, it has been concluded that the system should be fed in the right amounts for the new and refurbished products to increase the effectiveness of factors such as advertising, incentives, and environmental awareness for achieving the desired sales amounts and cost targets.

## Introduction

**Fig. 1:**
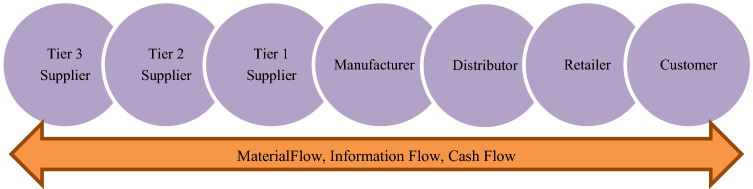
A generic forward supply chain network

Globalizing world conditions provide a great market opportunity to businesses. In 2020, the global supply chain management market was valued at 15.85 billion U.S. dollars and is expected to reach almost 31 billion U.S. dollars by 2026 [118]. To gain a competitive advantage, businesses give more importance to the design and management of their supply chain networks day by day. The supply chain network is a complex structure that consists of actors, such as suppliers, manufacturers, distribution centers, retailers, customers, and the flows between them (Fig. 1).

Interest of traditional forward supply chains starts with the process of acquisition of raw materials from suppliers and moves on to the production of products at manufacturers, and the delivery of products through distributors and retailers to customers. Examining, designing, and managing of this complex structure are crucial for the efficiency of businesses. In a 2020 survey, demand-side challenges, such as faster response time were cited among the most difficult hurdles supply chain companies face [118]. On the other hand, there are some catastrophic impacts of supply chain activities in terms of environment. Loss of biodiversity, deforestation, running out of raw materials, toxic waste, damage to ecosystems, hazardous emissions, and depleting landfill capacities are some of these impacts. According to the report by the World Wide Fund for Nature (WWF) until 1970 humanity was demanding resources and emitting carbon dioxide at a rate that the ecosystem within their borders could keep up with, however the situation has dramatically reversed in the last fifty years because of explosion in global trade, consumption and human population growth, as well as an enormous move towards urbanization. Humanity enterprise currently demands 1.56 times more than the amount that Earth can generate and an average 68% decline occurred in population sizes of mammals, birds, amphibians, reptiles, and fish between 1970 and 2016 [102].

Nowadays, there is a growing need to deal with the challenges made by supply chain operations on the global environment and control their negative impact [67]. Thus, with an increasing importance of sustainability, reverse logistics, ecological footprint concepts, businesses have started to incorporate reverse flows into their supply chain networks for several reasons, such as preventing environmental deterioration by reducing resource and energy consumption during industrial activities, generating less waste, meeting the expectations of conscious customers or stakeholders, and fulfilling the requirements of environmental protection and waste management laws obliged by governments. In addition to forward flow extending from supplier to customer, the systems dealing with reverse flow simultaneously for recovering or disposing properly of used products are called closed-loop supply chain (CLSC) networks. Reverse flow-specific facilities, such as collection centers, disassembly centers, repair centers, recycling centers, refurbishing centers, remanufacturing centers, and landfills may be located in CLSC networks for the efficient management of recovery and disposal of returns along with located centers in traditional supply chains. Efficient design and modeling of CLSC networks is crucial for the management of these centers and for both the forward and reverse flows between them, simultaneously.

### Closed-loop supply chain network design

**Fig. 2:**
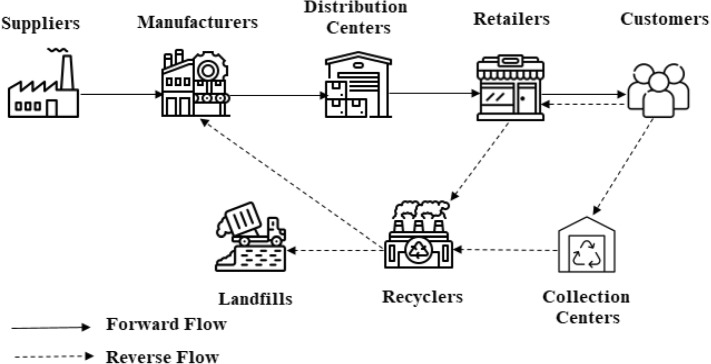
A closed-loop supply chain network for recycling

The supply chain network is, in its simplest form, the whole of all the connections that provide the forward flow of products between suppliers, manufacturers, distribution centers, and customers. After delivering to customers, because of end-of-life or end-of-use, warranty coverage, incorrect or defective delivery; products may return to the supply chain. After separation and inspection, there is a decision to perform one of the reverse logistics options, such as repairing, remanufacturing, refurbishing, recycling, and cannibalization, according to the quality of the returned product and technical and economic possibilities. According to the reverse logistics option to be applied, different amount of disassembly may be required. Recovery may not always be possible. In such cases, returned products are disposed of properly, in a way not to harm the environment and human health. It shouldn’t be forgotten that the origin of reverse flow in the supply chain does not always have to be customers. Reverse flow can also be triggered among the other members of the chain due to trade agreements, incorrect delivery, and product recalls. Systems that handle forward and reverse flows as a whole are called CLSC. A generic CLSC network for recycling option is given in Fig. 2.

Beside recycling different reverse logistics options can be performed to the products depending on their quality that was returned to the supply chain for one reason or another. Products that are returned to the chain can be directly reused without any operation or after minor operations if the condition of the product is good. In the case of mediocre quality for reusing, the returned product can be recovered by one of the repairing, refurbishing, remanufacturing, cannibalization, or recycling processes [3]. The returned product is disposed when technological or economic application of any of the recovery options mentioned above is not possible. One or more of these options can be considered together which will reduce the amount of waste, regarding the product characteristics.

Decision makers should cope with a lot of uncertainties such as in customer demands, in the quality, quantity, and time of the returns while designing and modeling the CLSCs. Since CLSC network design and modeling problems aim to optimize not only the flows between the forward facilities but also between the reverse facilities, these problems are more complex than the supply chain network design problems that merely considering forward and reverse flows. Furthermore, CLSC network design problems are in the class of NP-hard problems and the required time for solution of the analytical model is increased exponentially with the increase in the size of the problem.

### Agent-based modeling

Agent-based modeling (ABM) consists of autonomous agents that interact with each other and is used to model complex systems [59]. Since the agents in the agent-based system are in communication with each other and their environment, the ABM methodology is preferred for the solution of distributed and complex problems. Successful results can be obtained when ABM is used for problems involving the following situations [26]: (i) in large-scale problems, (ii) in cases that ask for a short time solution, and (iii) in dynamic problems. It is easier to reach a solution because the problem is divided into smaller local problems with agent-based approaches. In short time requirements, agents interact with their environment, can make individual decisions, and do not deal with very complex problems. Since the agents act independently, they do not have to wait for each other's decisions. Thus, the solution of problems can be reached quickly. Because ABM is suitable for changes, it is easy to add or remove agents into the dynamic system. Agent-based approaches have a high degree of interchangeability as it is relatively simple to delete or add agents when necessary throughout the study period.

Being autonomous, social skilled, real, cooperative, reactive, and changeable are the basic features of agents to be used in modeling a supply chain. Using ABM instead of the optimization technique for modeling CLSC network problems, some advantages can be achieved [27]. Firstly, when using optimization methods, the solution time for complex and large-scale problems can increase drastically. Conversely, large-scale problems are handled modularly because ABM divides the main problem to local problems. Besides, ABM can flex the assumptions required for analytic modeling so that strengthen the reality of the model. Lastly, agents can react quickly to changes as they can continuously monitor the situation of the local environment but optimization techniques often require a relatively long time to respond to these changes. Agent-based systems have attracted attention in recent years. There are studies using ABM for different types of problems. Some of them are given below.

Chen and Wang [97] developed a fuzzy dynamic-prioritization agent-based system to improve the forecasting of the cycle time of a job in a wafer fabrication plant.Colon et al. [23] formulated an ABM to explain the impact of a disaster on the transport-supply chain nexus. The model simulated the behavior of firms experiencing transportation and supply disruptions. Lu et al. [58] expanded a model to explore how individuals behave and the evolutionary mechanism of the life cycles using agent-based modeling. Ying and O’Clery [108] modeled virus transmission in supermarkets based on an agent-based model of customers. In the study, a simple virus transmission model was formulated based on the time a customer spends near infected customers. Marvuglia et al. [62] presented an ABM-Life cycle assessment model of agricultural production in the Grand Duchy of Luxembourg. The goal of the study was to evaluate the effects of farmers' interactions in their social networks on agricultural activities. Rahbar et al. [80] presented a hybrid approach consisting of ABM and deep learning algorithms to creating automated 2D architectural layouts.

In this study, a generic CLSC network consisting of supplier, manufacturer, distribution center, and customer for the forward network and collection center, disassembly center, and landfill for the reverse network was designed. Simultaneous forward and reverse flow of materials and products in this CLSC network were handled. In the proposed network, demands are considered in two ways, namely new product demand and refurbished product demand. Refurbished products are obtained by collecting the used products from customers at collection centers, performing the disassembly process to get valuable parts, equipping obtained parts with new technologies, repairing, updating, and using the parts in production. The demands of customers for new and refurbished products were handled dynamically. The high degree of uncertainties caused by the dynamic environment further complicates the already complex supply chain network. The solution time required for solving these types of problems via analytical models increases exponentially with increasing problem sizes. In this study, mostly ignored parameters when the analytical modeling approach is used for CLSC network design and modeling problems, such as time required for production, separation, disassembly operations, and economic life of products are included in the system with the intention of getting more realistic results through ABM methodology. Furthermore, various assumptions made for facilitating the solution via analytical modeling are eliminated using the ABM approach.

## Literature survey

In recent years, humanity has faced many threats such as the depletion of natural resources and increasing natural disasters due to the deterioration of the ecological balance. Because, environmental concerns of governments and customers have increased, the interest in CLSCs are getting attention in both academy and practice. For a CLSC that can compete in the global market successfully, effective supply chain network design is inevitable. There are many studies addressing CLSCs from different perspectives, such as network design and modeling, performance measurement, inventory management, and scheduling in the literature. Here, only CLSC network design and modeling studies are mentioned due to widespread literature. The first comprehensive studies on CLSC modeling in the literature are studies by Jayaraman et al. [47] and Fleischmann et al. [35]. Jayaraman et al. [47] presented a binary mixed integer programming model for obtaining the optimum quantities of transported and produced quantities and locations of remanufacturing/distribution centers. Fleischmann et al. [35] proposed a general location problem for the product recovery network. They compared the reverse logistics networks with traditional forward ones.

In addition to these studies, some of the comprehensive studies about CLSC network design and modeling are mentioned below. Pishvaee et al. [76] proposed a robust optimization model for handling the inherent uncertainty of input data in a CLSC network design problem. Amin and Zhang [8] examined a CLSC network containing multiple products, facilities, collection centers, and demand markets and proposed a mixed integer linear programming (MILP) model that minimizes total cost. The proposed model was solved by two ways including weighted sums and e-constraint methods. Demirel et al. [29] proposed a mixed integer programming model for a CLSC network with multi-periods and multi-parts considering two main policies as secondary market pricing and incremental incentive policies. In the study, using a realistic network example, several scenarios were generated and explored. The authors investigated the effects of various parameters such as demand, capacity, purchasing costs and size of the network on the performance of the problem. Rezapour et al. [82] proposed a bi-level model for strategic reverse network design and operational planning of a closed-loop, single-period supply chain operating in a competitive environment with price-dependent market demand. An existing supply chain comprising the production and distribution of new products, and a new competing supply chain with both new and remanufactured products was considered in the study. Özceylan et al. [71] presented CLSC network design and modeling for end-of-life vehicles treatment in Turkey. They developed a linear programming model to handle the reverse material flows with the aim of reintegrating them into forward supply chains. Several CLSC scenarios were discussed to show the performance of the proposed model and its applicability in the automotive industry. Paydar et al. [74] proposed a MILP model for the CLSC consisting of the collection and distribution of used engine oil. Robust optimization approach was used in the study to deal with the uncertainty in the amount of oil collected. Hajiaghaei-Keshteli and Fard [51] developed a new mixed integer non-linear programming model to formulate a multi-objective CLSC network design problem that considers the transportation cost reduction supposition. In the solution of the problem, in addition to traditional and current metaheuristic methods, the algorithms were used by hybridizing according to their strengths. Sahebjamnia et al. [85] developed a model for closed-loop tire supply chain network design considering economic, environmental, and social dimensions. In the study, the main advantages and disadvantages of individual algorithms were considered and four new hybrid metaheuristic algorithms were developed to solve large-scale problems. Yıldızbaşı et al. [107] developed a mixed integer programming model for automotive CLSC network design problem. Four different interactive fuzzy programming approaches were used in the study to tackle the trade-offs among the objectives. Fakhrzad and Goodarzian [33] presented a new fuzzy multi-objective programming approach for a production–distribution model for a multi-product, multi-period, and multi-level CLSC problem. The problem was formulated as multi-objective MILP model.

Goodarzian et al. [41] proposed a new multi-objective, multi-stage, multi-product, multi-period pharmaceutical supply chain network with the problem of production, distribution, purchasing, ordering, inventory, keeping, allocation, and routing under uncertainty. In the study, a new robust fuzzy programming method was developed. Isaloo and Paydar [45] aimed to improve performance in conditions of uncertainty associated with the plastic injection industry. Accordingly, the authors proposed a bi-objective mathematical programming model aimed at minimizing environmental emissions and total cost of the system including transportation costs, operating costs, and plant establishment fixed costs. Nayeri et al. [69] proposed a multi-objective mathematical model to configure a CLSC network for a water tank, considering sustainable measures. In the study, fuzzy robust optimization was applied to deal with the uncertainties of the CLSC network problem caused by changes in parameters such as transportation costs and demands. The proposed model was solved using goal programming approach. Pourmehdi et al. [78] developed a multi-objective linear mathematical model under uncertainty to optimize a sustainable CLSC for steel. The uncertainty was modeled in the stochastic environment and the proposed model was developed through a fuzzy goal programming approach. Abdi et al. [1] developed a new stochastic optimization model for the CLSC network design problem. Whale optimization algorithm which is a new nature-inspired algorithm and particle swarm optimization were used to address the model developed using a two-stage stochastic programming. Babaeinesami et al. [16], proposed a multi-objective model in the area of CLSC problem integrated with lot sizing by considering lean, agility and sustainability factors simultaneously. In the study, responsiveness, environmental, social and economic aspects were regarded. Strategic and operational backup decisions were developed to increase the resiliency of the system against disruption of the facilities and routes simultaneously. Biçe and Batun [19], considered the problem of designing a CLSC network including the uncertainty in demand quantities, return rates, and quality of returned products. They designed the problem as a two-stage stochastic mixed-integer program to maximize the profit of the system. Boronoos et al. [20], developed a multi-objective mixed integer nonlinear programming model for a closed-loop green supply chain network design problem. In the study, the objectives were minimization of the total costs, total CO_2_ emissions, and robustness costs in both forward and reverse directions, simultaneously. Chouan et al. [22], designed a multi-stage sugarcane supply chain network to process the by-products produced. In the study, three hybrid metaheuristic algorithms were proposed to handle the complexity of the problem and the performance of the algorithms was investigated using Taguchi experiments. Diabat and Jebali [30] studied the CLSC network design problem for durable products that can be disassembled into different components when they reach their end-of-life. Authors proposed models for designing the CLSC network based on various environmental legislation assumptions. The models were applied on a case study for washing machines and tumble dryers arising in Germany. Lotfi et al. [57] designed a CLSC by considering sustainability, flexibility, robustness, and risk aversion. In the study, robust counterpart model was used to handle uncertainties and a two-stage MILP model was proposed for the problem. Mosallanezhad et al. [65] presented a mathematical model for the shrimp supply chain network that optimizes the total cost of the entire network. In the study, three meta-heuristics were used to solve the shrimp supply chain network design problem composed of distribution centers, wholesalers, shrimp processing factories, markets, shrimp waste powder factory, and shrimp waste powder market. Pazhani et al. [75] developed strategic decision-making models for two different CLSCs with multiple products over multiple periods. Supply chain networks designed to assist decision making in terms of inventory, location and shipping were modeled using integer linear programming. Salehi-Amiri et al. [86] designed a CLSC network for the walnut industry by reviewing past studies. For the designed network, a MILP was developed that minimizes the overall costs. Yolmeh and Saif [109], proposed a mixed integer non-linear programming model for a CLSC network design problem integrated with assembly and disassembly line balancing under demand and return uncertainty. In the study, an enhanced decomposition approach was developed to solve the proposed model. Zahedi et al. [111] proposed a new CLSC network with sales agency and customers. The aim of the model, which had four echelons in the forward direction and five echelons in the backwards direction, was to maximize the total profit. The structure of the model was based on MILP and the proposed model was investigated through a case study regarding the manufacturing industry.

Akbari-Kasgari et al. [6], designed a copper network to reduce the effects of earthquakes on mining operations and used backup suppliers as a resilience strategy. In the study, multi-purpose models consisting of economic, environmental and social purposes, as with backup and without backup, were presented. Arabi and Gholamian [10] aimed to optimize the design of a closed-loop stone supply chain network. In the study, a multi-period multi-product mixed integer quadratic programming problem was considered. In the proposed model, two-stage stochastic programming was applied to address quality uncertainty in the mining supply chain taking into account the quality characteristics of the final stones. Kazancoglu et al. [50] aimed to present a multi-objective optimization model for a green dual-channel supply chain network that addresses economic and environmental issues. A MILP was proposed in a green dual-channel and CLSC network design. Kim and Chung [52] formulated a supply chain model to determine whether reverse logistics facilities, manufacturing centers, suppliers should relocate to their home country from the host country to maximize the total profit based on the level of reshoring drivers. The effects of productivity-adjusted costs and reverse logistics on the reshoring decisions were analyzed. Salehi- Amiri et al. [87] formulated a CLSC network for the avocado industry by developing a bi-objective model. In the study, the costs of the avocado industry and the social factor of job employment opportunities were discussed within the framework of total cost minimization and employment maximization objectives. Tavana et al. [94] designed an integrated multi-objective MILP model to design sustainable CLSC networks. In the model, cross-docking, location-inventory-routing, time window, supplier selection, order allocation, transportation modes with simultaneous pickup, and delivery under uncertainty were considered. Tirkolaee et al. [96] developed a multi-objective mathematical model to design a multi-period, multi-echelon, multi-product, sustainable mask CLSC network during the COVID-19 outbreak. In the study, a MILP model was proposed to address the locational, supply, production, distribution, collection, quarantine, recycling, reuse, and disposal decisions. Apart from the above studies, there have been many studies in the field of CLSC network design and modeling in the literature [9, 11, 28, 42, 44, 53, 56, 68, 77, 79, 88, 99, 103, 104, 106, 115, 116]. In some of the studies, exact solution algorithms were used for solving the problems [13, 28, 42, 44, 77, 79], as well as heuristics and meta-heuristics solution methodologies [11, 14, 34, 68, 96, 107], and simulation techniques [37, 72, 92, 95]. To reach comprehensive studies on CLSC network design modeling, readers may refer to the works of [7, 32, 43, 46, 91, 100].

Supply chains have a complex structure composed of many components. The most important of these components is customers, the only source of revenue for the supply chain. Since customer behavior directly influences the profitability and cost of the network, it needs to be addressed in detail in the supply chain network design. In the literature, studies of [15, 24, 66, 70, 84] considered the customer behavior in supply chain networks. Only in the study of [69], customer behavior had been considered in the reverse logistics network design problem. In the study, the authors proposed a goal-programming model to design a green supply chain network by dividing the customers into three segments according to their green expectations.

Although there are several studies dealing with the issues of ABM and supply chain together in the literature [2–5, 12, 17, 18, 21, 25, 27, 36, 38–40, 48, 49, 54, 55, 60, 61, 63, 64, 73, 81, 83, 89, 90, 98, 101, 105, 110, 112–114], the use of ABM methodology in supply chain network design and modeling is much less when compared to other areas, such as scheduling, capacity planning, and production planning [3]. Furthermore, very few of these studies have handled reverse flows with ABM technique. Golinska et al. [39] discussed the fundamental problems arising from the simultaneous material flow planning in the CLSC. The authors proposed a model for the integration of CLSC through an agent-based system. Yang and Wang [104] proposed an ABM approach to overcome the difficulty of predicting reverse logistics activities due to the constraint of information transparency. In a tutorial titled “How to build a combined agent-based system dynamics model in AnyLogic”, an example was given, in which customers were defined as agents. In the example, customers' demand behaviors for products were considered agent-based [117]. Golinska [40] investigated specific tools that allow efficient management of the information accompanying the material flows in a CLSC and presented a theoretical background. The author discussed the potential for implementing agent-based solutions to improve reverse logistics management. Mishra et al. [64] proposed a multi-agent model to overcome the complexity of recycling used products and related logistics management issues. The authors handled waste classification, recycling, logistics, and reuse of products in the proposed agent architecture to assist manufacturing industries in efficient management of their green supply chains. Sauvageau and Frayret [88] proposed an agent-based simulation model to analyze the performance of various procurement and production policies in the cyclic paper industry. The authors introduced a procurement behavior model considering both the marketing price and the inventory requirements in collaboration for a large pulp maker that was also performing recycling operations in North America. Authors also presented a waste paper marketing model, imitating the market price and controlling the validity of price forecasting. Pandia et al. [73] used ABM to measure the performance of a reverse logistics company. The authors identified each member of the reverse logistics network as an agent and ensured each member measures their own performance. Since the behavior of customers has a direct impact on the profitability of the supply chain, it is necessary to consider customer behavior in network design. There are few studies that consider customer behavior in supply chain network design. Although there have been few existing studies analyzing the effect of customers’ behavior on supply chains or performing the ABM approach for managing forward or reverse logistics network design, to the best of the authors’ knowledge, there is no study that focuses on the impact of customers’ behavior on the design and modeling of CLSCs and uses the ABM approach for managing forward and reverse flows, simultaneously. In this study, to fill this gap in the literature, a generic design of dynamic CLSC network that deals with forward and reverse flows was proposed. The ABM method was used with the aims of integrating customer behaviors into the network, coping with the dynamic customer arrivals, and obtaining a more realistic structure by eliminating the assumptions of the model. Demands were classified for new and refurbished products in the proposed model. The effects of parameters, such as advertising, other users’ experiences, and market incentive threshold values on customer purchasing behavior were taken into consideration. On the other hand, it is obvious that lead time grows into a more critical factor in customers’ decision making. For example, in the UK, 65% of automobile customers pointed out the waiting time from order to delivery has a significant effect on their decisions to buy a new car; moreover, 61% of car buyers like to receive their car within 14 days or less [31, 93]. Therefore, in the case of customers not finding the product that they preferred (new product/refurbished product) in the distribution centers, the possibility of changing the preference and purchasing another type of product and resulting penalty costs were also taken into consideration.

The applicability of the proposed model was demonstrated in an example. The effects of different parameters to the network were analyzed via several scenarios. The main contributions of this research are to deal with the computational complexity associated with solving the large-scale supply chain network design problems using analytical modeling techniques as well as taking real-life conditions into account while designing CLSC networks. The novel aspects of the research can be listed as follows: (i) to develop a generic CLSC network design model focusing on customer behaviors (ii) to consider dynamic customer arrivals (iii) to eliminate required assumptions for solving such a problem via analytic methods using ABM (iv) to take into account the production, separation, and disassembly times, and economic life of the new and refurbished products (v) to involve quantity, time, and quality uncertainties of returns, and (vi) to present different scenarios according to different parameter settings to observe the behavior of the supply chain network for different conditions.

## Agent-based modeling for closed-loop supply chain network design

### Structure of the problem

**Fig. 3:**
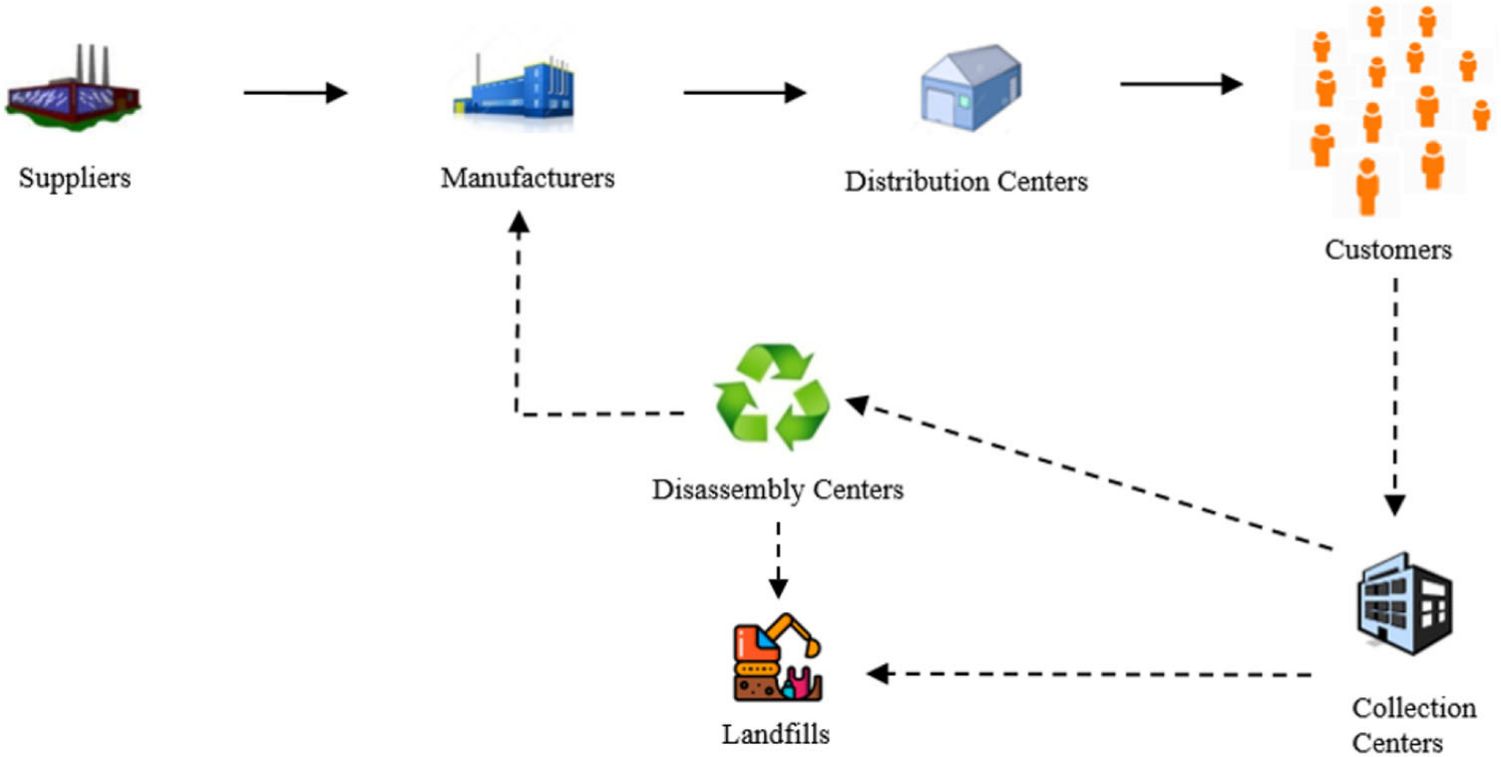
The structure of proposed closed-loop supply chain network

In CLSC networks including forward and reverse flow simultaneously, forward flow begins with the purchase of raw materials and/or parts from suppliers and finishes with the delivery of goods to the customers. Customers are not only the endpoint of the forward flow, but also the starting point of the reverse flow. The collection of end-of-life/end-of-use products from customers at collection centers is the first activity of reverse logistics. The reverse flow ends with the returned products being treated and reintroduced into the forward network or disposed of in landfills. The aim of the problem examined in the study was the efficient management of both forward and reverse flows by designing a generic CLSC network using the ABM approach. While managing flows, the dynamics of customers’ demands for new and refurbished products and the impact of these dynamics on the behavior of the CLSC network were considered. In this context, all supply chain actors were defined as agents and information about how each agent acts for each purpose were processed. The proposed CLSC network, including the forward and reverse actors is given in Fig. 3.

In the proposed network, new products are produced using cores that are received from suppliers and refurbished products are produced using obtained parts through the disassembly process and come from disassembly centers to the manufacturers. The new and refurbished products meet the customers’ demands through the distribution centers. Due to reasons, such as obsolescence of technology, degradation, and completion of economic life, unwanted customer products are collected at collection centers. Products collected in collection centers are inspected and separated. Good quality products are sent to disassembly centers and others are sent to the landfills for disposal. Used products coming to disassembly centers are disassembled here. Obtained reusable parts are sent to manufacturers to use in the production of refurbished products and the residue is sent to landfills for disposal. Collected products that do not meet quality standards are properly disposed in landfills together with useless residue coming from disassembly centers. To gather a more realistic model, dynamic arrivals of customers to the system, preference of customers for waiting for the requested product type or purchasing which is available in stock are considered. In addition, external factors that affect customers' product type selection are taken into account in the model.

The proposed CLSC network has been modeled with ABM to incorporate various dynamics into the network. The actors in the CLSC network are defined as agents and the objectives, characteristics, and behaviors of each agent are determined. Utomo et al. [98] defined producer agent, post-harvest agent, processor agent, retailer agent, consumer agent, other agent in their study. Backs et al. [17] defined manufacturer agents and consumer agents in their approach. In this study, supplier agent, manufacturer agent, distribution center agent, customer agent, collection center agent, disassembly center agent, landfill agent were determined. The information about the agents and their properties are given below:

*Supplier agent* This agent meets the demand of manufacturers for parts within the agent’s capacity. If the agent does not have enough capacity, the agent interacts with the related manufacturer, as soon there is capacity.

*Manufacturer agent* This agent demands cores from the nearest supplier with the aim of meeting the demand of new products of the distribution center. If the capacity of the supplier is not sufficient, the manufacturer agent gets in contact with the second nearest supplier. If none of the suppliers has enough capacity, the manufacturer will wait until the cores are provided from the suppliers. When the cores reach the manufacturer, production of new products is actualized and the produced batch is transported to the relevant distribution center. The manufacturer agent also checks the stock of reusable parts obtained from returned products in disassembly centers to meet the demand of refurbished products of the distribution centers. When there are enough parts, production of refurbished products is actualized or waits until the disassembled parts arrive.

*Distribution center agent* This agent meets new and refurbished product demands of customers from center inventory. When the new and refurbished product inventories decrease to a predetermined reorder level, the agent orders a predetermined batch size from the manufacturers. When the products arrive at the distribution center, the relevant customer, waiting for an order, is notified. New and refurbished product demands are met separately.

*Customer agent* This agent may prefer a new product under the influence of advertisements and other users or by their own choice. In addition, the agent may prefer a refurbished product depending on environmental awareness, other users’ experiences, or the market incentive threshold value performed for refurbished products. In the model, to incorporate the dynamism in customer preferences into the system, a random value is produced and purchasing behavior of customers is changed depending on whether the produced value is above or below a market incentive threshold value. The customer agent purchases the new or refurbished products from the closest distribution center that has the product in inventory. In case any distribution center has no inventory to meet the demand of new or refurbished products, the customer agent may choose to wait according to an assigned patience rate or demand another quality product (the demand of new product may switch to refurbished product and vice versa). Depending on the randomly determined life span of the product, the agent may choose to bring the unwanted product to the nearest collection center or not to bring it anywhere after using it. Lastly, the demand of an agent may arise again when the life of its product ends or the agent may leave the system because this type of product is no longer wanted.

*Collection center agent* This agent inspects returned products from customers and transports good quality products to the disassembly centers and the remaining to the landfills.

*Disassembly center agent* This agent disassembles the returned products coming from the collection center in good quality and stores the obtained parts to be sent to manufacturers. The agent, which operates as a pushing system, transports the parts to the manufacturers when they reach a certain batch size. Residues are sent to landfills for disposal.

*Landfill agent* This agent properly disposes of products not satisfying the quality conditions and residues of disassembly centers.

The structure of the problem and assumptions are given below:

- The production of new and refurbished products is actualized with the same manufacturer.
- The reorder levels of distribution centers for new and refurbished products and the ordering batch sizes from the manufacturers are predetermined and known.
- Refurbished products are cheaper than new products.
- The economic life of new products is longer than the economic life of refurbished products.
- In case a customer cannot find the demanded type of product (new or refurbished) in the distribution centers, he may wait until the product arrives or may decide to purchase another type. If the customer prefers another type instead of waiting, the system is subjected to a predetermined penalty cost.
- Inventory cost is ignored.

**Fig. 4:**
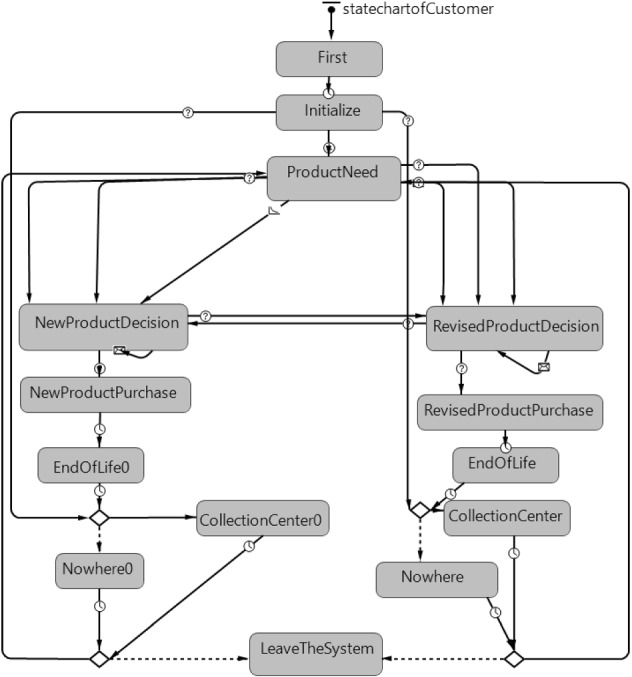
Statechart of customer agent

**Fig. 5:**
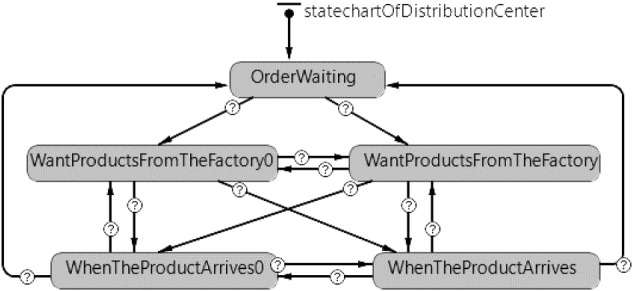
Statechart of distribution center agent

**Fig. 6:**
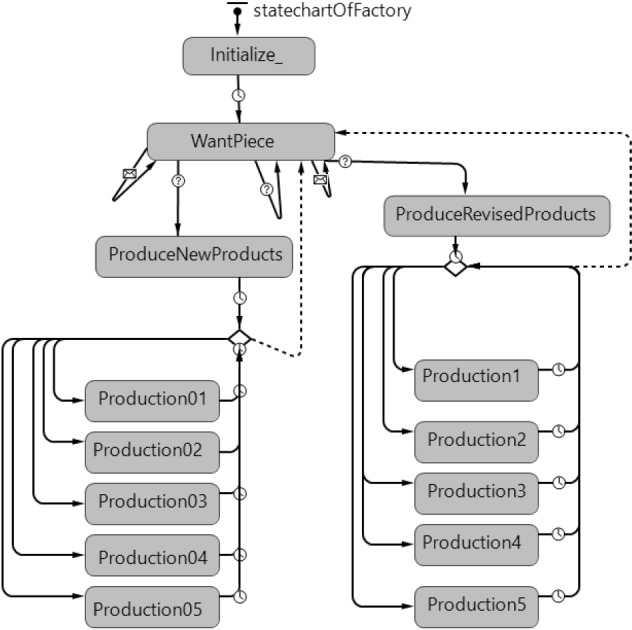
Statechart of manufacturer agent

**Fig. 7:**
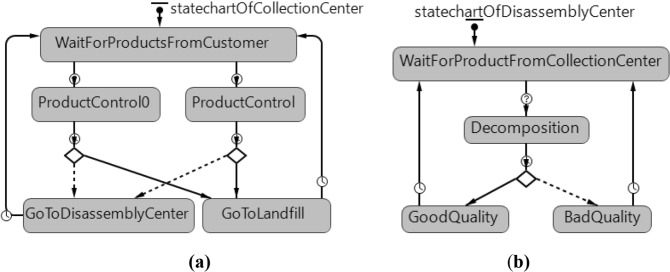
**a** Statechart of collection center agent. **b** Statechart of disassembly center agent

The statecharts defining the behaviors of the agents are given in Figs. 4, 5, 6 and 7.

In the statechart given for the customer agent in Fig. 4, the customer enters the system dynamically according to a certain arrival rate (the rate is the frequency of the event per unit time) and goes into the ‘Initialize’ state. The customer entering the system gets location and patience rate values that are randomly generated. The customer agent then goes to one of the ‘ProductNeed’, ‘CollectionCenter’, or ‘Nowhere’ states. A customer agent that goes into the ‘ProductNeed’ state may choose to buy new product due to the transition that indicates its own preference. The customer agent may also choose to buy a new product due to the transition for being affected from advertisements or the transition for being affected by other users’ experiences. A customer agent choosing to purchase a new product enters the ‘NewProductDecision’ state. When an agent is triggered by environmental awareness reasons, market incentive threshold value, or other users’ experience transitions, the agent may choose to buy a refurbished product. Customer agents that prefer a refurbished product will switch to the ‘RevisedProductDecision’ state. Customers in one of the ‘NewProductDecison’ or ‘RevisedProductDecision’ states purchase the product from the closest distribution center. If there is no product in the closest distribution center, the agent looks to other distribution centers in order of proximity. In case of not finding the desired type of product in the distribution centers, it may stop waiting according to the patience rate and prefer another quality type of the same product. When the customer agent no longer wants a product that has been purchased, the agent may choose to bring the product to the collection center at the end of the period determined for life span transition or not return it to the system. When the agent chooses to return the product, the product is brought to the closest collection center and enters one of the ‘CollectionCenter0’ or ‘CollectionCenter’ states according to whether the agent has purchased a new or refurbished product at the beginning of the process. If the agent chooses not to return the product to the system, the agent passes to one of the ‘Nowhere0’ or ‘Nowhere’ states according to whether the agent has purchased a new or refurbished product at the beginning of the process. The probability of bringing back a customers’ new and refurbished product to the collection centers differs due on the quality status of the products (The probability of return of new purchased products to the system after using is higher than refurbished ones). Customers intending to repurchase a product pass to ‘ProductNeed’, otherwise it passes to the ‘LeaveTheSystem’ state.

The flow for the distribution center agent is given in Fig. 5. Customer orders are met from the inventory of distribution center. When the number of products in the distribution centers drops to the reorder point, it changes from the ‘OrderWaiting’ state to the ‘WantProductsFromTheManufacturer0’ state for the new product and to the ‘WantProductsFromTheManufacturer’ state for the refurbished product. When the ordered batch arrives at the distribution center, it passes to the ‘WhenTheProductArrive0’ state and the ‘WhenTheProductArrive’ state for the new and refurbished products, respectively. With the delivery of the obtained products to the relevant customer, the distribution center agent returns to the ‘OrderWait’ state.

A manufacturer agent passes from ‘Initialize’ state to the ‘WantPiece’ state when the demand for a new product comes from a distribution center and core parts are demanded from the closest supplier (Fig. 6). Along with acquiring core parts, the manufacturer agent passes to the ‘ProduceNewProduct’ state. Production is carried out by staying in this state during the production period. When a demand of distribution centers for refurbished products is actualized, the manufacturer agent goes to the ‘WantPiece’ state from the ‘Initialize’ state, similarly. Production is carried out by switching to the ‘ProduceRevisedProduct’ state. These parts are obtained by the manufacturer through the disassembly process in the disassembly centers via a push system. Production is made by staying in this state during the production period. To ensure that there are no distribution center orders to be produced, the agent returns to the ‘WantPiece’ state after checking the distribution centers’ new and refurbished product orders.

Figure 7a gives a collection center agent statechart. Returned products from customers are waited in the ‘WaitForProductsFromCustomer’ state. As soon as products arrive to the collection center, the agent assigns the ‘ProductControl0’ and ‘ProductControl’ according to the type of the brought product whether it is new or refurbished. In this state, the inspection of the returned products is carried out during the control period. At the end of the period, the products go to the ‘GoToDisassemblyCenter’ state for to obtain reusable parts through the disassembly process or to the ‘GoToLandfill’ state for disposal forbad quality. The agent updates the inventory level of the relevant center and returns to the ‘WaitForProductsFromCustomer’ state.

Figure 7b gives a statechart of a disassembly center agent. The flow starts with the transportation of collected products from the collection centers to a disassembly center. The agent’s ‘WaitForProductFromCollectionCenter’ state changes to ‘Decomposition’ state. The disassembly process is carried out during the determined period and a decision is made according to the quality condition of the part at the end of the disassembly process. The agent is assigned ‘GoodQuality’ state or ‘BadQuality’ state, depending on the quality of the obtained part. Parts that satisfy quality standards are sent to manufacturers, while the remaining are sent to landfills.

### An illustrative example

The proposed problem has solved for an example consisting of 2 suppliers, 1 manufacturer, 5 distribution centers, 3 collection centers, 1 disassembly center, 1 landfill, and several customers that enter the system dynamically. A small set of parameters reflecting a real-life industrial case has been selected for the example. Unit transportation cost is assumed to be 0.2 in terms of monetary unit per kilometer and unit purchase cost of parts from the suppliers is taken as 2 monetary units. Unit production cost for new and refurbished products is assumed to be the same and considered as 7 monetary units. The collection, disassembly, and disposal costs of the used products are determined as 2, 5, and 3 monetary units, respectively. Capacities of all suppliers are taken as the same, as 50 units per week. The other parameters used in the model are given in Table 1.

**Table 1:** Parameters of the model

	New product	Refurbished product
Selling price (monetary unit)	120	80
Life cycle (year)	Uniform [2, 3]	Uniform [1, 2]
Probability of return	0.5	0.4
Reorder point for distribution centers (unit)	40	20
Batch size of distribution centers(unit)	50	25
Number of parts obtained by disassembly	2	2
Unit production time (minute)	30	30
Quality satisfaction probability	0.8	0.6

In the model, customers arrive in the system dynamically, according to uniform [2, 12] distribution (hours). A customer entering the system locates according to the *x*, *y* coordinates that has assigned to them regarding uniform [0, 1000] distribution. When customers cannot find the desired type of product in terms of quality (new or refurbished), a value of 0.1, 0.4, or 0.6 is assigned to the customers as the patience rate, referring to giving up waiting and preferring other qualities of the same product (i.e., preferring a new product instead of a refurbished one and vice versa). The customer may arrive to the system for purchasing a new or refurbished product or only to return a used product. The probability that a customer entering the system has come to drop a used product at a collection center is taken as 0.05. The rate of customers affected by advertising is assumed as 1 per day and the rate of customers being affected by other users’ experiences as 0.5 per day. The probability of demanding a new product with their own preference is taken as 0.4. Customers may prefer to purchase refurbished products because of other users’ experience with the rate of 0.5, or because of their environmental awareness with the rate of 0.3. When returning a used product to the system, the probability of a repeated customer’s demand is considered as 0.9. In the collection center, the required time for inspection of returned products is set to be 0.1 h per unit. For returned products coming to the disassembly center, unit disassembly time is determined as 0.4 h and 2 parts are assumed to be obtained through the disassembly process. Parts obtained through the disassembly process are sent to manufacturers with a probability of 0.8.

The model considers that customers' preferences are affected depending on the market incentive threshold value applied for the refurbished products. As the market incentive threshold value decreases, the probability of demand by customers for refurbished products increases. Similarly, high environmental awareness increases the demand for refurbished products, whereas the increase in the rate of exposure to advertisements increases the demand for new products.

### Computational results and scenario analyses

The developed model is run for different scenarios according to different parameter settings to observe the behavior of the supply chain for different conditions and to analyze the effectiveness. Different scenarios are developed depending on the changes in the capacities of the suppliers, changes in the market incentive threshold values, and changes in customer behaviors. The model is run 10 times for each scenario. 16 different scenarios given in Table 2 are developed and in total, 160 problems are solved.

**Table 2:** Developed scenarios for analyzing the behavior of the model

Scenario	The rate of customers being affected by advertising	Environmental awareness of customers	Capacity of suppliers (unit/week)	Market incentive threshold value
1	1 in day	Low	100	1.5
2	1in 2 days	Low	100	1.5
3	1 in day	High	50	1.5
4	1in 2 days	High	50	1.5
5	1 in day	Low	50	1.5
6	1in 2 days	Low	50	1.5
7	1 in day	High	100	1.5
8	1in 2 days	High	100	1.5
9	1 in day	Low	50	2
10	1in 2 days	Low	50	2
11	1 in day	High	50	2
12	1in 2 days	High	50	2
13	1 in day	Low	100	2
14	1in 2 days	Low	100	2
15	1 in day	High	100	2
16	1in 2 days	High	100	2

**Fig. 8:**
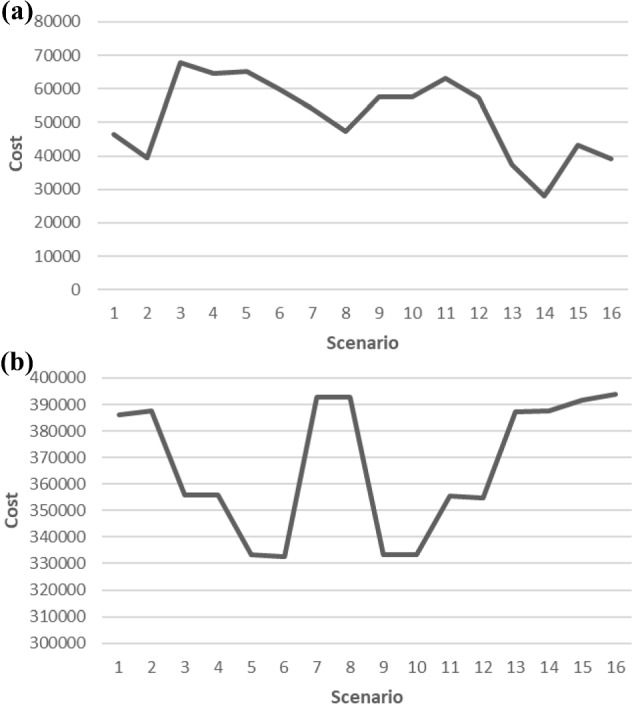
**a** Average total penalty cost, **b** Average total production cost

All runs of the problem are done on a server with 2.7 GHz Intel Core i7 processor with12 GB of RAM and using ANYLOGIC-PLE software for a total of 15 years, including a 3-year warm-up period. When customers prefer other types of products instead of waiting, the system is subjected to a penalty cost. Figure 8a shows the average total penalty cost incurred by the system for different scenarios. The highest average total penalty cost is actualized in scenario 3, while the lowest cost is seen in scenario 14. These results can be interpreted as below:

- In scenario 3, due to the high rate of customers being affected by advertising, the demand for new products has been increased. In the case of insufficient supplier capacities, the average number of customers giving up waiting for the new product has been actualized as 3945, which has caused an increase in the average total penalty cost.
- In scenario 3, due to the increase in environmental awareness of customers and decrease in market incentive treshold values, the number of customers demanding refurbished products have been increased as expected. Despite this, it can be seen that the average number of customers giving up waiting for a refurbishing product is actualized as 18,679.6, which has increased the average total penalty cost.
- In scenario 14, the minimum average total penalty cost has been actualized because there is no customergivingup waiting to purchase a new product. Moreover, the average number of customers, who gives up waiting to purchasing a refurbished product, has been actualized as 9374, which is about half the value realized in Scenario 3.

**Fig. 9:**
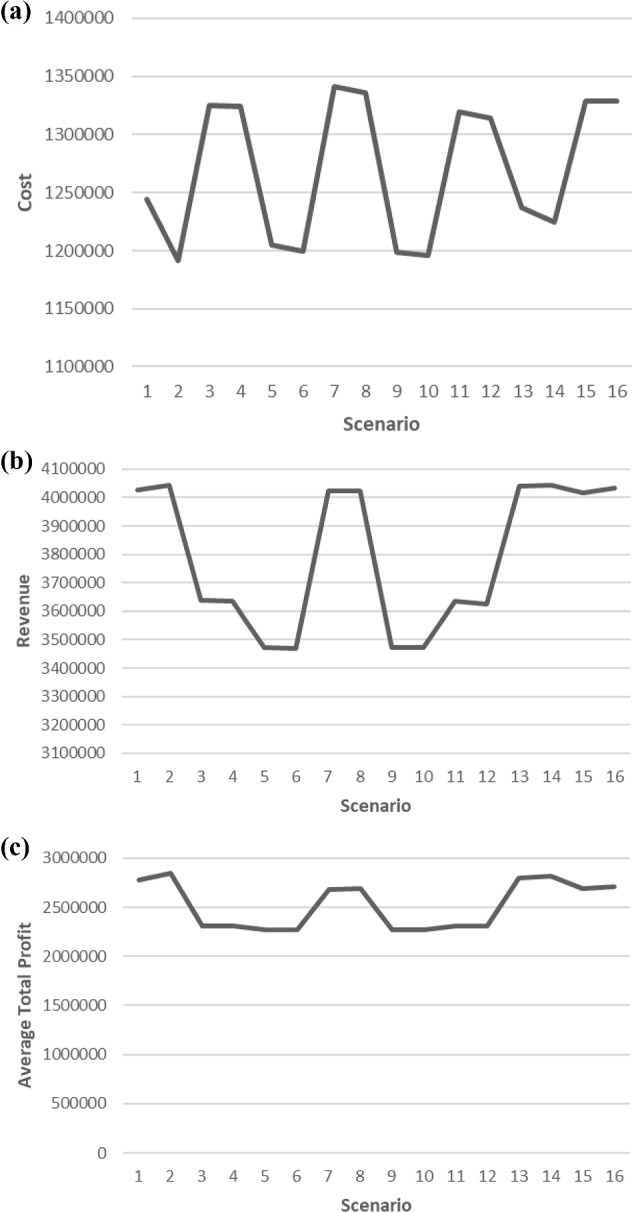
**a** Average total cost. **b** Average total sales revenue. **c** Average total profit

It can be seen from Fig. 8b that the line representing the average total production cost is parallel with the line representing the average total sales revenue given in Fig. 9b. This result shows that more production, thanks to increased supplier capacities, means more sales revenue when compared with other scenarios. It can be interpreted as the most important point affecting sales is the feeding of the production and that advertisements have less effect on sales than supplier capacities do. It can be concluded from Fig. 9a that scenario 7 has the highest average total cost whereas scenario 2 has the lowest one. The highest average total profit in Fig. 9c has been occurred at the point when the minimum average total cost and highest average total sales revenue occurred. In Scenario 2, which has the highest average total profit (Fig. 9c), the average number of new products that has been sold is 42480, and this number has been actualized as 12,908 for refurbished products. In Scenario 14, similar results are obtained with Scenario 2 for average total profit, average total cost, and average total sales revenue. Since other parameters are the same, except for incentive rate, it can be concluded that the market incentive threshold value don’t have a significant effect on average total cost and average total profit. On the other hand, in Scenario 2 the average number of customers giving up refurbished products is actualized as 13135.5, when getting 9374.3 in Scenario 14.

Table 3 shows the average values of cost and profit components for all scenarios. In Table 4, the average values of the parameters that affect profit and cost in each scenario are given.

**Table 3:** Average values of cost and profit

Parameters (Average)	1	2	3	4	5	6	7	8	9	10	11	12	13	14	15	16
Total production cost	386,190	387,590	355,827.5	355,775	333,305	**332,710**	392,840	392,787.5	333,287.5	333,287.5	355,530	354,742.5	387,415	387,765	391,807.5	*393,890*
Total collection cost	42,445.4	42,491	51,522	51,593.2	40,979.4	**40,865.6**	51,854	52,078.8	41,094.6	41,094.6	51,571	51,562.6	42,441.2	42,451	51,941.4	*52,107.6*
Total disassembly cost	80,659.5	80,793.5	96,814	96,898	77,837	**77,531.5**	97,148.5	97,686	78,036.5	78,036.5	96,775.5	96,715	80,540	80,748.5	97,558.5	*97,758*
Total disposal cost	24,827.1	25,001.1	30,834	30,921	24,148.2	**24,132.3**	31,104.3	*31,267.5*	24,252.9	24,252.9	30,913.5	30,948.6	24,969.6	24,887.1	31,039.5	31,194.3
Total penalty cost	46,355.1	39,406.5	*67,873.5*	64,485.6	65,219.4	59,930.7	53,971.2	47,303,7	57,478.8	57,478.8	63,205.5	57,195.6	37,425.9	**28,122.9**	43,122.3	39,150
Total sales revenue	4,027,292	4,043,815	3,638,381	3,636,174	3,473,618	**3,467,700**	4,024,324	4,025,220	3,471,969	3,471,969	3,633,340	3,626,147	4,040,870	*4,044,951*	4,015,567	4,035,133
Total piece purchasing cost	168,920	*170,000*	141,800	141,720	141,160	141,000	162,320	161,960	**140,960**	**140,960**	141,680	141,160	169,960	169,880	161,560	162,640
Total transportation cost	*746,690.4*	**396,861.5**	580,248.2	582,328.5	522,366.1	523,700.8	551,595	552,581.7	523,660.4	521,123	580,440.1	581,786.7	494,822.9	490,988.3	551,346.4	552,242
Total cost	1,244,515	**1,191,491**	1,324,919	1,323,721	1,205,015	1,199,871	*1,340,833*	1,335,665	1,198,771	1,196,233	1,320,116	1,314,111	1,237,575	1,224,843	1,328,376	1,328,982
Total profit	2,782,777	*2,852,324*	2,313,462	2,312,453	2,268,603	**2,267,829**	2,683,491	2,689,555	2,273,198	2,275,736	2,313,224	2,312,036	2,803,295	2,820,108	2,687,191	2,706,151

The values specified in bold are the minimum values in the row, and the values written horizontally are the maximum values in the row

**Table 4:** Display of average values of some parameters which are effective on profit and cost

Parameters (Average)	1	2	3	4	5	6	7	8	9	10	11	12	13	14	15	16
Number of new products sold	42,247.4	42,480.0	35,803.2	35,767.8	35,654.6	**35,602.5**	40,585.3	40,552.5	35,604.3	35,572.0	35,748.0	35,665.9	42,451.5	*42,487.7*	40,431.4	40,660.6
Number of refurbished products sold	12,950.0	12,908.3	15,482.5	15,495.0	12,425.0	**12,390.0**	15,550.0	15,620.4	12,472.5	12,415.0	15,470.0	15,457.5	12,895.0	12,918.7	15,621.1	*15,645.7*
Number of customers giving up new products	**0**	**0**	3944.9	4248.8	4340.2	4508.3	**0**	**0**	4432.6	*4518.9*	4117.3	4373.1	**0**	**0**	**0**	**0**
Number of customers giving up refurbished products	15,451.7	13,135.5	*18,679.6*	17,246.4	17,399.6	15,468.6	17,990.4	15,767.9	14,727.0	12,026.8	16,951.2	14,692.1	12,475.3	**9374.3**	14,374.1	13,050.0

The values specified in bold are the minimum values in the row, and the values written horizontally are the maximum values in the row

Results have shown that the average total transportation cost has increased in scenarios where suppliers had low capacities. Table 3 shows minimum total sales revenue and minimum total profit for scenario 6. Although costs are generally low in scenario 6, there is no significant difference compared to other scenarios. Maximum total sales revenue is observed in scenario 14. However, maximum total profit is observed in scenario 2. This is due to the total minimum cost is observed in scenario 2. Scenario 6 differs from scenarios 2 and 14 because the supplier capacity of scenario 6 is low. The difference between scenario 14 and scenario 2 is the value of the market incentive threshold value. The market incentive threshold value in scenario 2 is higher than scenario 14. Table 4 shows the average number of sold new and refurbished products and the number of customers who have changed their preference. It can be seen from the table that the points with the highest amount of average new products sold pertain to scenarios 1, 2, 13, and 14. The common point of these scenarios is the higher supplier capacities. The parameter of being affected by advertisements can be interpreted as not as effective as supplier capacities on the behavior of the supply chain.

The highest average number of refurbished products sold has actualized in Scenarios 8 and 16 (Table 4). When these two scenarios are examined, it is concluded that the increase of the environmental awareness affects the demand of refurbished products positively, as expected. In these scenarios, the increase in the demand of refurbished products is higher than the increase in the average number of refurbished products sold due to the high average number of customers who give up waiting for the refurbished products. This situation indicates that there are not enough refurbished products in the market and it can be interpreted as the system should be fed with refurbished products in accordance with increasing environmental awareness.

According to the scenario analyses, the prominent managerial insights can be listed as follows: (i) feeding the production is an important issue to obtain more sales revenue, since the foremost factor for the sales revenue is determined as supplier capacities, (ii) the strategies to collect returned products are important, because the sales amount is not enough depending on the insufficient supply of refurbished products in the system although the factors such as environmental awareness and market incentives affect the demand of refurbished products positively, (iii) Scenario 2 is the most profitable scenario in the presented experimental design. In this scenario, value of the rate of customers being affected by advertising, environmental awareness of customers, capacity of suppliers, and market incentive threshold are 1 in two days, low, 100, and 1.5, respectively. The other two scenarios following the second scenario in terms of profitability are the Scenarios 13 and 14, (iv) Since the amount of refurbished products in the system generally is not enough to meet the demand, the cost is increasing due to unmet refurbished product demand. The common striking point in these three profitable scenarios is that the factors triggering to purchase the refurbished products are mostly at their low levels.

As seen in the analyses, the proposed model is a promising approach for the managers who struggle to manage their CLSCs in the dynamic and stochastic environment. With the development of technologies that enable to monitor the data in systems instantaneously such as RFID systems and their use in supply chains, managers can obtain instantaneous data of the systems. It is important to make decisions by considering these data to compete with the other supply chains. In many real-life problems, dynamic changes in the forecasted demand affect the supply chain plans that are often done via analytical methods. It is often not possible to resolve these problems and obtain new plans in every dynamic change in the system with analytical methods. Even if it is resolved, every new solution affects the entire system. Therefore, with each solution, it may be necessary to restructure the operations in all the processes. This is often not possible or too costly. Managers can make dynamic decisions with the agent-based modeling approach, which allows to include online data to the solution without resolving the problem, and offers effective solutions without disturbing the whole system. In today's competitive environment including frequent dynamic changes, such a decision support system is very important for managers. In the proposed system, customers enter the system dynamically. The scenario analyses showed that the proposed agent-based method can handle these dynamics efficiently. In addition, many parameters that affect the demands are defined and customer demands are shaped by agent-based modeling. Similarly, the amount of recovery is determined by modeling the behavior of the customers and incorporated to the system. Here, most of the parameters are considered stochastically as in many real-life problems. Thus, we hope that this approach will support the managers about demand forecasting, determination of important parameters of the dynamic system, and effective management of the supply chain.

## Conclusion

The advantages of applying green supply chain activities in terms of conservation of natural resources, energy savings, and reduction of environmental damage have increased the importance given to CLSC networks and effective management of the forward and reverse flows in these networks.

One of the most difficult issues in supply chain management is to manage the changes in customer demands. The proposed model handles the dynamic customer arrivals so as the demands in the system. In the proposed model, customer demands are obtained according to the defined customer behaviors via agent technology. Uncertainty uncertainty in the time, quality, and quantity of returns increases the complexity of the supply chains incorporating reverse flows compared to traditional supply chains and makes it more difficult to manage them effectively. The demands depend on several factors and are shaped by the customer features, behaviors, and interactions among themselves. By the proposed ABM architecture, it was aimed to design and model a generic CLSC network, considering dynamics inherent in supply chains and all actors involved in the supply chain have been identified as agents. The applicability of the proposed model is shown via scenario analyses. By the analyses, the effects of the changes in the rate of advertisement, capacities of the suppliers, market incentive threshold values, and customer behaviors are examined. In the proposed approach we also considered various parameter values as stochastic, such as returning rates of end-of-life products, qualities of returned products, and life cycles for different types of products to obtain more realistic model. The demonstration of the proposed model on an example and scenario analyses for different parameter settings showed that the model can effectively cope with the dynamic and stochastic character of the data, especially customer behaviors. In this context, the developed model presents an effective decision support system for supply chain managers in today's dynamic work environment.

The success of the ABM technique in handling the dynamic structure, individual goals and behaviors into the model is very suitable for the nature of the supply chain. The proposed model is especially helpful in managing the difficulties in modeling supply chain networks that include reverse flow, which are more complex than forward flow networks. The proposed approach provides to the companies a network management opportunity that is highly understandable and highly visual, successful in reflecting reality, and able to follow the changes instantly. The developed ABM architecture can be improved by considering transportation times, vehicle occupancy rates and storage costs as future research. The ABM technique can be used for multiple products or for competitor manufacturers. Furthermore, the preference of the customer agent in choosing the closest collection and distribution center can be modeled with the proposal mechanism and results can be compared. Today, besides the environmental and economical aspects, social issues are also getting attention as one of the main trivets of sustainable supply chain management. Therefore, proposed model can be improved for supply chain network design in which social factors are taken into account. Lastly, the proposed approach can be applied to a real-world problem.
